# Differential Structural Development of Adult-Born Septal Hippocampal Granule Cells in the Thy1-GFP Mouse, Nuclear Size as a New Index of Maturation

**DOI:** 10.1371/journal.pone.0135493

**Published:** 2015-08-12

**Authors:** Tijana Radic, Omar Al-Qaisi, Tassilo Jungenitz, Marcel Beining, Stephan W. Schwarzacher

**Affiliations:** Institute of Clinical Neuroanatomy, NeuroScience Center, Goethe-University Frankfurt, Frankfurt am Main, Germany; University of Nebraska Medical Center, UNITED STATES

## Abstract

Adult neurogenesis is frequently studied in the mouse hippocampus. We examined the morphological development of adult-born, immature granule cells in the suprapyramidal blade of the septal dentate gyrus over the period of 7–77 days after mitosis with BrdU-labeling in 6-weeks-old male Thy1-GFP mice. As Thy1-GFP expression was restricted to maturated granule cells, it was combined with doublecortin-immunolabeling of immature granule cells. We developed a novel classification system that is easily applicable and enables objective and direct categorization of newborn granule cells based on the degree of dendritic development in relation to the layer specificity of the dentate gyrus. The structural development of adult-generated granule cells was correlated with age, albeit with notable differences in the time course of development between individual cells. In addition, the size of the nucleus, immunolabeled with the granule cell specific marker Prospero-related homeobox 1 gene, was a stable indicator of the degree of a cell's structural maturation and could be used as a straightforward parameter of granule cell development. Therefore, further studies could employ our doublecortin-staging system and nuclear size measurement to perform investigations of morphological development in combination with functional studies of adult-born granule cells. Furthermore, the Thy1-GFP transgenic mouse model can be used as an additional investigation tool because the reporter gene labels granule cells that are 4 weeks or older, while very young cells could be visualized through the immature marker doublecortin. This will enable comparison studies regarding the structure and function between young immature and older matured granule cells.

## Introduction

Adult neurogenesis is a process in which new neurons are generated from neural stem cells (NSCs) in the adult brain. In the adult hippocampus of mammals, including humans, dentate granule cells (DGCs) are continually generated in the subgranular zone (SGZ) and settle within the granule cell layer (GCL) of the dentate gyrus [1–4]. Although the majority of adult-born DGCs in rodents die within the first month [5,6], the surviving cells become structurally and functionally integrated into the existing cellular network and thus contribute to hippocampus-dependent functions involving learning, memory, and emotion [7–9]. More specifically, these neurons appear to play an essential part in spatial memory and pattern separation [8,10–12]. Dysfunction of the adult neurogenesis process has been linked to neurological and psychiatric diseases, including epilepsy, Alzheimer's disease, and depression [13]. Detailed understanding of developmental processes and mechanisms involved in adult neurogenesis is fundamental to enable therapeutic strategies for neuronal loss and brain repair [13,14].

Growth and maturation of newly born neurons in the adult hippocampus show much similarity to the embryonic development of DGCs [3,15]. However, adult-born DGCs seem to mature at a slower pace [16,17] and need several weeks or longer to become functionally integrated [18–20]. There are still open questions in relation to the time course of development and functional activity of adult-generated DGCs, as some studies appear to generate contradicting results regarding the involvement of newborn DGCs in the existing cellular network (for review see [20,21]). This could be due to a high variability in the neuronal developmental course and the regulating factors involved in it. In order to fully understand the developmental process and characteristics that are necessary for DGCs to become integrated into the hippocampal network, a more detailed examination of the cells' maturation process is essential.

Detailed structural information of neurons can be acquired in the transgenic Thy1-GFP mouse model in which the reporter gene GFP is expressed in approximately 10% of all DGCs [22]. It has been demonstrated that labeled cells do not differ in morphology or function compared to DGCs that do not express Thy1-GFP [23]. In the present study, we show that the Thy1-GFP mouse model could be used to investigate the structure of DGCs beyond the maturation phase and thus enable comparative studies of mature and newly-generated DGCs.

Recently, we have demonstrated that the speed of structural development varies substantially between individual adult-born DGCs in the rat [19]. We introduced a 6-stage classification system of structural maturation based on morphological characteristics of cells that express the immature neuronal marker doublecortin (DCX). In the current study, we adapted the staging method to closely examine the structural development of adult-generated DGCs in mice and investigate the relationships between structural maturation and age, as well as cell position and nuclear size. Our results reveal a general correlation between structural development and age as well as a considerable variability in growth dynamics between individual cells.

In addition, we found that the size of a cell's nucleus is indicative of its age and degree of structural maturation and could therefore be used as an additional parameter for cell development. Hence, our detailed evaluation of granule cell morphological maturation provide a structural basis and novel techniques for future studies of adult-born DGC development and integration into the mature hippocampal network.

## Materials and Methods

### Animals

Adult male Thy1-GFP transgenic mice (GFP-M line, see [22,23]; 18–26 g) were bred on a C57BL/6 background and housed under standard conditions at the animal facility of Goethe University Frankfurt, Germany, in a 12 h light/dark cycle (lights on at 0600 hours), with food and water available *ad libitum*. Animal care and experimental procedures were performed in agreement with the German law on the use of laboratory animals (animal welfare act; TierSchG; §4 par 3) and approved by the animal welfare officer of Goethe-University, Faculty of medicine (reference number BB01/10/2011).

### BrdU administration

In order to label newly born cells, 5-bromo-2´-deoxyuridine (BrdU; AppliChem) was administered to each animal with a single intraperitoneal injection (200 mg/kg body weight) at postnatal day 42 (P42). Animals were sacrificed at 7, 14, 21, 28, 35, 49, 63, and 77 days post BrdU injection (dpi) for evaluation of newborn neuron survival at these time points.

### Immunohistochemistry

Animals were anesthetized with isoflourane and transcardially perfused with 0.9% NaCl followed by 4% paraformaldehyde (PFA) in 0.1M phosphate buffered saline (PBS). Brains were removed, postfixed in 4% PFA overnight at 4°C, and serially sectioned in the frontal plane (75 μm) with a vibratome. Sections were washed in TRIS-buffered saline (TBS; pH 7.40), and stored in cryoprotectant solution containing 30% ethylenglycol and 25% glycerin in 0.1M PBS at -20°C. Free floating sections were washed in TBS and incubated in a blocking solution containing 5% bovine serum albumin and 2.5% Triton X-100 for 1 hour at room temperature. In stainings involving BrdU detection, sections were pre-treated with 2 N HCl for 30 minutes at 37°C to denature DNA, followed by neutralization in 0.1M boric acid (pH 8.5) for 10 minutes at room temperature, and three rinses in TBS prior to blocking. Subsequently, sections were incubated in the primary antibody solution containing 1% Triton X-100 and 2% BSA in 0.1M TBS for 48 hours at room temperature. The following primary antibodies were used against: BrdU (rat, polyclonal, 1:250, Abcam, Ctl. A-2139), doublecortin (DCX, goat, polyclonal, 1:500, Santa Cruz, Ctl. SC-8066), and Prospero-related homeobox 1 gene (Prox1, rabbit, polyclonal, 1:1000, ReliaTech, Ctl. 102-PA30S). For visualization, sections were treated with secondary fluorescent dye conjugated antibodies (1:1000; Alexa 488, 568, and 633, Vector Labs., Burlingame, CA, USA) for 48 hours at room temperature.

### Histological data analysis

All data were acquired from three animals per age group (n = 3; at 7, 14, 21, 28, 35, 49, 63, and 77 dpi) and 3 sections per animal. High resolution (1024 x 1024 pixel) confocal images of histological frontal sections were obtained with a confocal laser scanning microscope (Nikon Eclipse 80i) using a 40x oil immersion lens (N.A. 1.3). Serial frontal brain sections containing the dorsal part of the hippocampal formation were collected. Series were rostro-caudally standardized according to the atlas of Franklin and Paxinos (1997). The first section corresponded to the stereotaxic position -1.265 mm from bregma and the last section corresponded to -3.065 mm from bregma [24]. For the Prox1, BrdU immunostaining, the 2nd, 11th and 20th serial sections were selected (corresponding to -1.34 mm, -2.015 mm, and -2.69 mm from bregma respectively), while the 7th, 16th and 25th sections (-1.715, -2.39, and -3.065 mm from bregma, respectively [24]) were used for triple immunostainings (DCX, Prox1, and BrdU).

In each section, three adjacent, non-overlapping regions of interest (medial, middle and lateral), were chosen for imaging along the suprapyramidal blade of the right dentate gyrus starting directly laterally from the crest where the supra- and infrapyramidal blades clearly separate. Image z-stacks (45–55 images per stack; z-axis interval between consecutive frames: 1 μm) were oriented perpendicular to the longitudinal axis of the granule cell layer (GCL). Each image frame had a width of 318.25 μm, i.e. the three frames together represented a total GCL length of 954.75 μm per section. Accordingly, cell counting in three sections per animal was always standardized against a total GCL length of 2864.25 μm. The GCL width, measured perpendicular to the longitudinal axes of the GCL, was found to be 53.363 ± 1.34 μm. Cell numbers for BrdU/Prox1+ and DCX+ cells were calculated per mm GCL length. DCX/BrdU/Prox1+ cells were counted in the same manner and standardized against the total number of BrdU/Prox1+ cells. The number of BrdU/Prox1+ cells at each time point was also standardized against the number of BrdU/Prox1+ cells at 1 week to determine the percentage of loss of newborn cells over time.

The degree of structural maturation of DCX+ cells was determined by classifying cells into one of six stages based on dendrite morphology (see [19]). Each stage was defined by the length and elaboration of the dendritic tree in the following manner: stage 1: the DCX+ cell soma is positioned in the subgranular zone (SGZ) and no processes are visible; stage 2: the DCX+ cell soma has one or two short processes that do not extend beyond the SGZ; stage 3: the principal dendrite of the DCX+ cell extends into the inner half of the GCL; stage 4: The leading dendrite reaches the outer half of the GCL; stage 5: the leading dendrite extends into the inner molecular layer (IML); stage 6: the leading dendrite extends into the outer molecular layer (OML) of the dentate gyrus. DCX+ cells that showed cut dendritic branches, i.e. labeled branches that reached the surface of the section, were excluded from analysis.

Nuclear size was determined by measuring the largest cross-sectional area of each Prox1+ cell nucleus using the polygon selection tab in ImageJ (Image Processing and Analysis in Java, version 1.43u). To determine predictive values of the nuclear size in relation to structural (DCX) stage and age of each granule cell, nuclear size data from different animals were pooled and categorized into an early and a late structural stage and cell age, considering DCX stage 1–3 and 7–14 dpi as an early phase and DCX stage 4–6 and 21–77 dpi as a late phase. The mean nuclear sizes of each phase were determined and used to calculate the equidistance between early and late phases, which was then used as a threshold to discriminate between early and late phases. Based on that threshold, single cells were classified into 3 different types of predictive values: true positive, false negative and false positive. The definitions for the different predictive values of the early phase were the following: true positive assigned cells belonged to the early phase and were below the threshold; false negative assigned cells belonged to the early phase, but were above the threshold and false positive assigned cells belonged to the late phase, but were below the threshold for the early phase and therefore assigned wrongly to the early phase. The conditions for the assignments of the late phase were defined as the opposite to the early phase.

The position of granule cell somata in relation to the SGZ and GCL was obtained by measuring the distance perpendicular to the medio-lateral axis of the GCL from the SGZ/GCL border to the lower border of the newly born granule cell’s nucleus which was normalized to the total distance from the SGZ/GCL border to the border of the GCL toward the IML (representing GCL thickness) at the particular confocal plane.

All statistic evaluations were performed with ≥ 3 animals (n ≥ 3) per group. For the correlation evaluation between nuclear size and DCX stage, data of all animals from 7 dpi to 28 dpi were pooled for analysis. Statistical differences between groups (stage 1: n = 5; stage 2: n = 7; stage 3: n = 4; stage 4: n = 6; stage 5: n = 9; stage 6: n = 11; n represents number of animals) were assessed with the non-parametric Kruskal-Wallis one-way analysis of variance (Dunn’s multiple comparison) test. Statistical significance was set at P < 0.05. Results are expressed as means ± SEM. All statistical analyses and graphs were produced using GraphPad Prism 5.03.

## Results

### Doublecortin-labeling in dentate granule cells does not co-localize with GFP expression in the Thy1-GFP mouse

**Fig 1:**
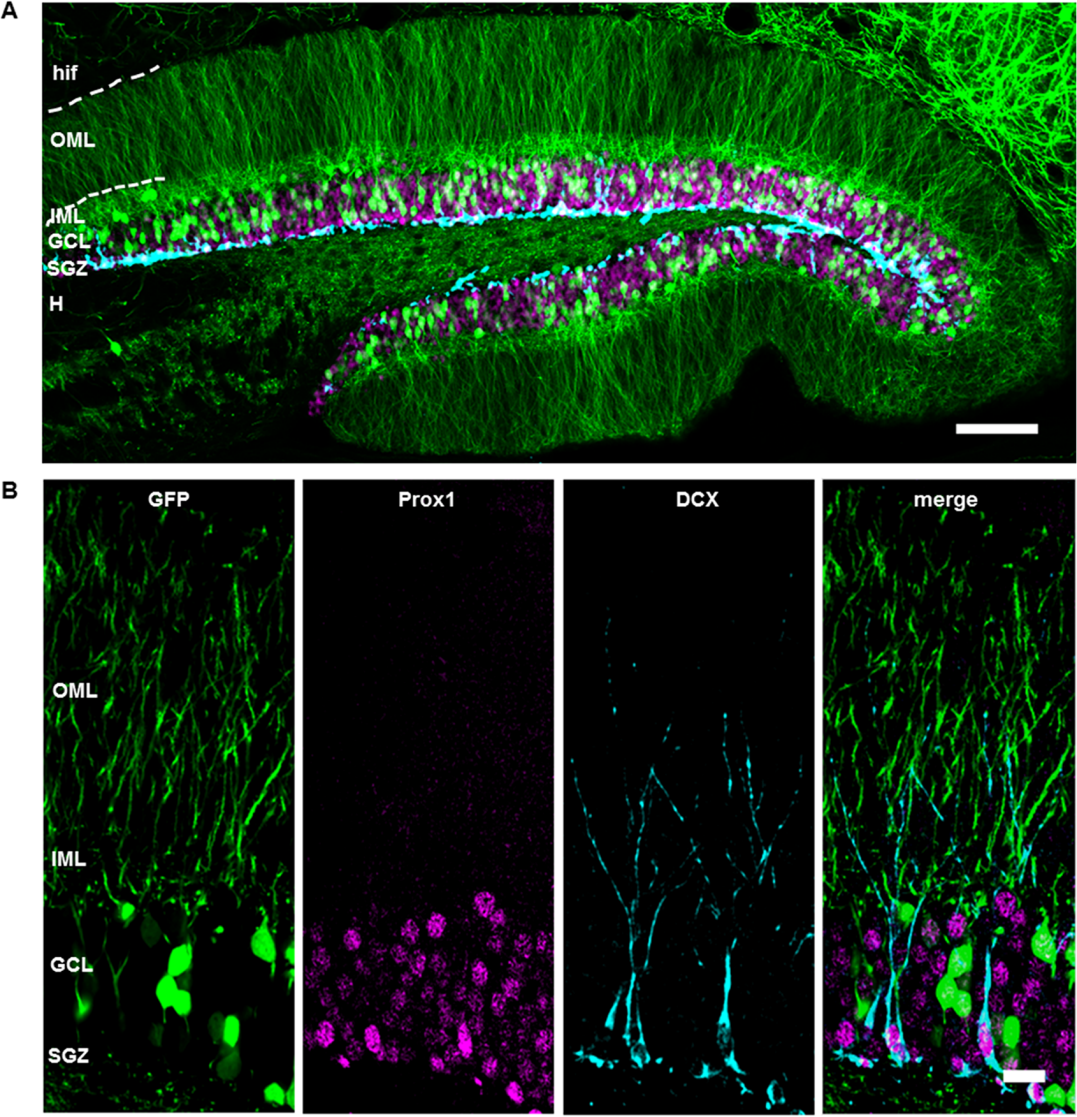
Doublecortin-labeling does not co-localize with Thy1-GFP expression. (A) Frontal section of the dorsal hippocampal formation from a Thy-1-GFP mouse. Thy1-GFP expression was observed in a subpopulation of dentate granule cells (DGCs) and was expressed throughout dendritic processes of DGCs which extend into the inner molecular layer (IML) and outer molecular layer (OML) toward the hippocampal fissure (hif). Prospero homeobox protein 1 (Prox1, magenta), a specific nuclear marker of granule cells, was confined to granule cell nuclei of the granule cell layer (GCL). Doublecortin (DCX, cyan) labeled young maturing cells that are positioned in the subgranular zone (SGZ). (B) There was no co-localization of DCX and Thy1-GFP which suggests that Thy1-GFP is generally expressed in more mature (DCX-) DGCs. Both DCX+ and Thy1-GFP+ granule cells co-localized with Prox1 even during early stages of DCX expression (see small DCX+ cells in the SGZ). Scale bars: (A) 100 μm; (B) 20 μm. CA1, Cornu Ammonis area 1; H, hilus.

In the present study, adult neurogenesis was investigated in the transgenic Thy1-GFP mouse model in which approximately 10% of all dentate granule cells (DGCs) express GFP. These cells presumably do not differ in morphology or function compared to unlabeled DGCs [23]. In order to evaluate to what extent Thy1-GFP might be present in maturing adult-born DGCs, we performed immunocytochemistry with doublecortin (DCX), a marker for immature neurons [25,26] and Prox1, a specific granule cell marker that is expressed early on and throughout the development of DGCs [27–29]. Prox1 was present in all detectable granule cell nuclei throughout the entire granule cell layer (GCL) and in the DCX+ cells. The somata of DCX+ cells were concentrated in the subgranular zone (SGZ) with dendritic processes extending toward the molecular layer (ML). In addition, Thy1-GFP expression was observed in a subset of DGCs, but there was no co-localization of Thy1-GFP and DCX. All Thy1-GFP+ cells of the GCL also expressed Prox1 (Fig 1). Even though Thy1-GFP and Prox1 are shown in the same color, the Thy1-GFP labeling was clearly distinguishable from the Prox1 staining, as Thy1-GFP intensely labeled the whole cell including the soma and dendritic processes as opposed to Prox1 which only labels the cell nuclei. The combination of these two markers in the same color channel did not pose any problems during analysis.

The first Thy1-GFP/BrdU+ cell was detected at 28 dpi. Overall, we found that 4.86% ± 2.48 (n = 15 animals) of BrdU-labeled cells between 28 and 77 dpi were Thy1-GFP positive. Taken together, our data indicate that, in the present mouse model, Thy1-GFP expression is only present in maturated DGCs. This offers the possibility to perform structural studies on two non-overlapping populations of immature DCX+ DGCs and mature Thy1-GFP+ DGCs in the same object simultaneously.

### Adult-born dentate granule cells show limited survival rate

**Fig 2:**
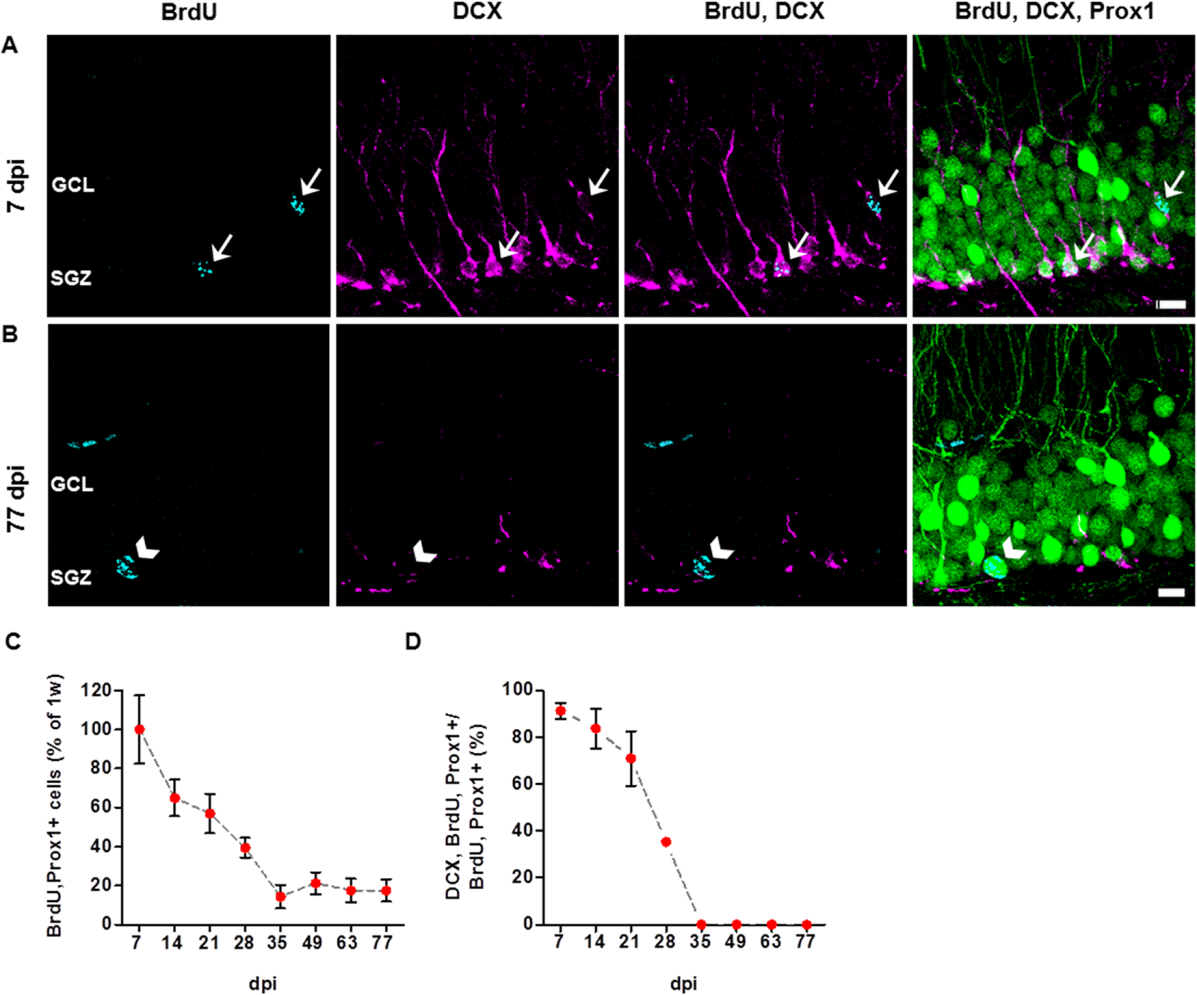
Survival rate of newborn dentate granule cells decreases over the first 4 weeks. (A, B) Newly born DGCs labeled with the mitosis marker BrdU (cyan) frequently displayed DCX-expression (magenta) at 7 days post BrdU injection (7 dpi; A), while there was no co-localization of BrdU and DCX at 77 dpi (B). All of the counted BrdU+ cells were Prox1+ (green; A, B). Arrows in (A) point to BrdU/DCX/Prox1+ cells. Arrowheads in (B) point to a BrdU/Thy1-GFP+ cell. Due to their intense somato-dendritic labeling, Thy1-GFP+ cells could be easily distinguished from the green nuclear Prox1 immunostaining. (C) Quantification of BrdU/Prox1+ cells revealed a decline in survival of newborn DGCs between 7 and 35 dpi, whereas the total number of BrdU+ cells did not change between 35 and 77 dpi. Compared to the first week post BrdU injection, 14% of BrdU/Prox1+ cells were retained at 35 dpi, after which there was no further cell loss. (D) The number of BrdU/Prox1+ DGCs that expressed DCX also decreased between 7 and 35 dpi. No DCX/BrdU/Prox1+ cells could be detected between 35 and 77 dpi. All analyses were performed in the suprapyramidal blade of the right dorsal dentate gyrus (n = 3 animals for each group, 3 sections per animal). Error bars represent SEM. Scale bars in (A, B): 10 μm. GCL, granule cell layer; SGZ, subgranular zone.

Next, we wanted to determine the proliferation and survival rates of newborn DGCs and, in particular, the population of DCX+ cells. The mitotic marker BrdU was administered to adult Thy1-GFP mice in order to label newborn cells. Co-immunostainings against BrdU, as well as the granule cell marker Prox1, and the immature neuronal marker DCX were performed between 7 and 77 days following BrdU injection. Fig 2A and 2B shows examples of newborn DGCs labeled with BrdU at 7 and 77 dpi, respectively. We calculated the number of BrdU+ DGCs as a percentage of the number of BrdU/Prox1+ cells at 7 dpi (Fig 2C). Our results reveal that the number of newly born neurons continuously decreased until 35 dpi (14.36% ± 6.04; see Fig 2C). At 14 dpi there were 64.89% ± 9.32; at 21 dpi, 56.91% ± 10.15; and at 28 dpi, 39.36% ± 5.07 BrdU+ cells. After 35 dpi, the number of newborn DGCs remained constant until 77 dpi.

Similarly, there was a steady decline in the number of DCX+ newborn DGCs between 7 dpi (91.32 ± 3.49%) and 28 dpi (35.39 ± 1.06%). From 35 dpi on, no BrdU/DCX+ cells could be detected (Fig 2E). This suggests that during the first 5 weeks after birth only a fraction of newly generated DGCs are selected for survival and eventual functional integration. Those that do survive undergo a process of maturation beyond the phase of DCX-expression.

We did not find Thy1-GFP expression in BrdU+ cells from 7–21 dpi. At later time points (28–77 dpi), co-labeled Thy1-GFP/BrdU+ DGCs were occasionally found, at very limited numbers unsuitable for further analysis. This indicates that Thy1-GFP expression may be present from day 28 in aging DGCs.

### Structural maturation of Doublecortin-expressing dentate granule cells increases with cell age

**Fig 3:**
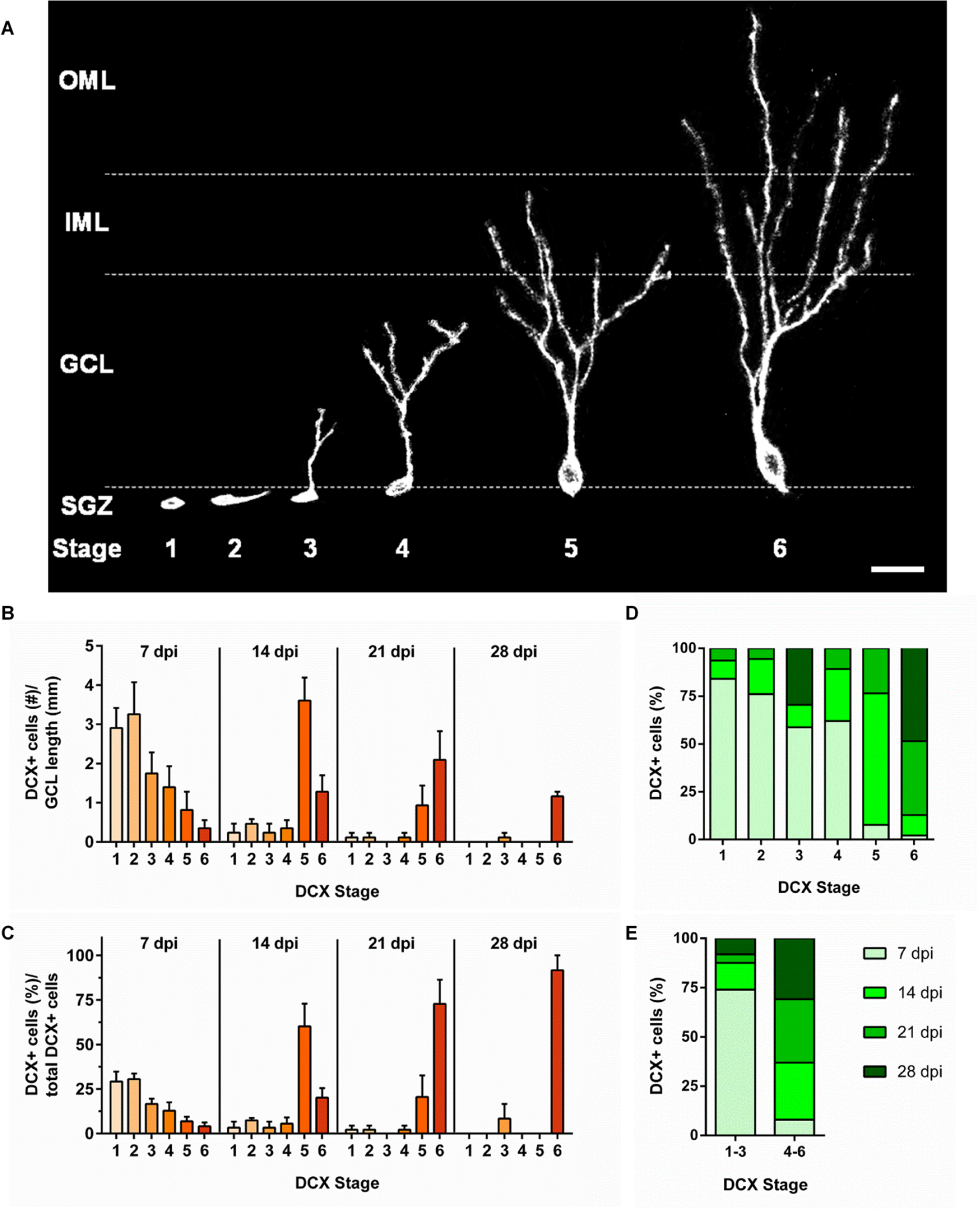
Structural maturation of DCX-expressing newborn DGCs is correlated with cell age. (A) DCX+ cells were categorized into six stages according to the degree of their structural maturation. Cells were considered to be in stage 1 when the soma was positioned in the subgranular zone (SGZ) and no dendritic processes were visible; stage 2 when the cell displayed short processes that were located within the SGZ; stage 3 when the principal dendritic process projected into the inner half of the granule cell layer (GCL); stage 4 when the leading dendrite reached the outer half of the GCL; stage 5 when the leading dendrite extended into the inner molecular layer (IML); and stage 6 when the leading dendrite reached the outer molecular layer (OML). (B, C) Staging of newborn DCX+ DGCs at different time points revealed a marked shift in stage distribution according to cell age. At 7 dpi, the majority of newborn DCX+ DGCs were classified as stage 1 or 2, while at 14 dpi, the majority of cells were classified as stage 5 or 6. At 28 dpi, about 92% of the DCX+ cells were classified as stage 6, but a small percentage of DCX+ cells were classified as stage 3. (D, E) The distribution of DCX+ cell ages according to each stage illustrates the prevalence of DCX stages 1–4 at 7 dpi and DCX stages 5–6 at 14 to 28 dpi. No BrdU/DCX+ DGCs were observed after 28 dpi. Notably, DCX+ cells of stages 1–6 co-existed at the same time points (7–21 dpi), suggesting a variability in the maturation time course of individual neurons. All data were obtained from 3 animals (n = 3) per group, and 3 sections per animal. Error bars represent SEM. Scale bar in (A): 20μm.

In order to analyze the degree and age dependency of structural maturation of newly formed DGCs, we further examined DCX/BrdU+ newborn DGCs at different time points between 7 and 28 dpi, i.e. the phase of DCX-expression. We adapted a DCX staging system that was previously established in the rat [19] to our mouse model. DCX+ cells were categorized into six different stages based on the extent of dendritic arborization and soma position. A DCX+ granule neuron was classified as stage 1 when its soma was located in the SGZ and lacked dendritic processes. When a cell exhibited one or two short processes that were located within the SGZ, it was considered to be stage 2. Once the principle dendrite of a cell extended into the inner half of the GCL, the cell was categorized as stage 3. Stage 4 was determined when the leading dendrite of the cell reached the outer half of the GCL; stage 5 when the leading dendrite extended into the inner molecular layer (IML); and stage 6 when the leading dendrite extended into the outer molecular layer (OML; see Fig 3A).

We found that the degree of structural maturation increased with cell age in the Thy1-GFP mouse model (Fig 3B and 3C). At 7 dpi, a substantial number of DCX+ cells were grouped in stage 1 (29.21 ± 5.57%) or stage 2 (30.56 ± 3.10%), while only a small number of cells were in stage 5 (6.83 ± 2.57%) or stage 6 (4.05 ± 2.12%). In contrast, most DCX+ granule neurons at 14 dpi were classified as stage 5 (60.20 ± 12.67%) or stage 6 (20.17 ± 5.31%). At 21 dpi, the vast majority of DCX+ DGCs were at stage 5 (20.56 ± 12.03%) or stage 6 (72.78 ± 13.62%). By 28 dpi, 91.67 ± 14.43% of the DCX+ cells were at stage 6. However, at this time point, we also found cells at stage 3 (8.33 ± 8.33%). The distribution of DCX+ cell ages according to each stage demonstrated the prevalence of DCX stages 1–4 at 7 dpi (DCX stage 1: 84.02%, stage 2: 76.09%, stage 3: 58.71% and stage 4: 62.06%) and DCX stages 5–6 at 14 (DCX stage 5: 68.74%, stage 6: 10.69%), 21 (DCX stage 5: 23.47%, stage 6: 38.58%), and 28 dpi (DCX stage 6: 48.59%; Fig 3D). DCX stages 1–3 and 4–6 were pooled and revealed that 72.02% of DCX+ cells of stages 1–3 were at 7 dpi, whereas DCX+ cells of stages 4–6 were essentially evenly distributed between 14 (28.96%), 21 (32.19%), and 28 dpi (30.88%; Fig 3E). No DCX+ cells were observed after 28 dpi.

Generally, the findings suggest that the degree of structural maturation of DCX+ cells increases with cell age. However, since we identified cells of all structural maturation stages at 7, 14, and 21 dpi, there appeared to be marked differences in the course of maturation between individual neurons within this time frame.

### Nuclear size of newborn DGCs is positively correlated with structural maturation and cell age

**Fig 4:**
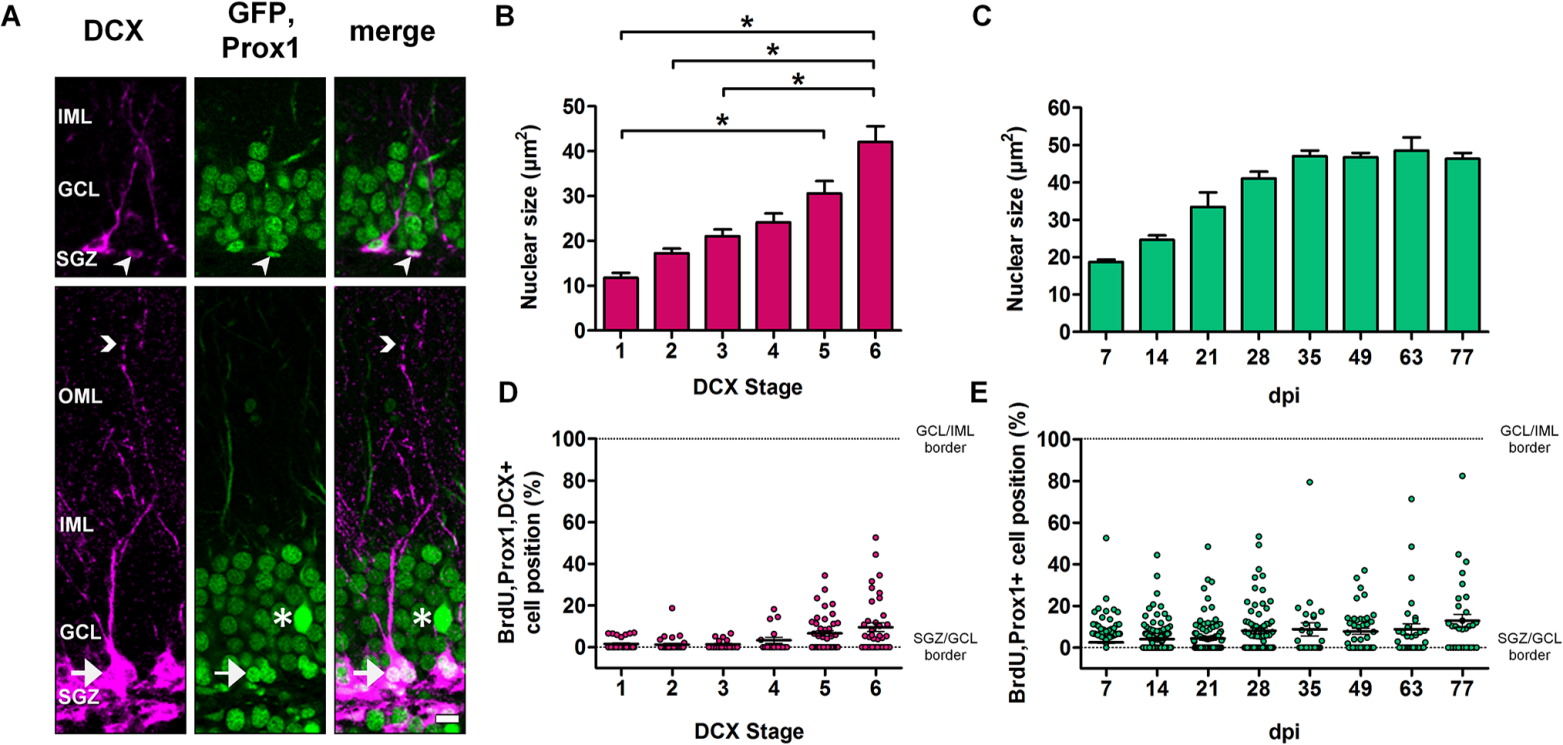
Nuclear size and soma position are positively correlated with structural maturation and age of newborn DGCs. (A) Examples of a 21-day-old stage 1 DCX/Prox1+ cell that is located in the subgranular zone (SGZ) and has no dendritic processes (arrowheads, upper panel) and a 21-day-old stage 6 DCX/Prox1+ cell (arrows, lower panel) with a dendrite extending into the outer molecular layer (OML; arrowheads, lower panel). Asterisk denotes the soma of an intensively labeled Thy1-GFP+ cell. (B) Nuclear size (determined with the nuclear marker Prox1, green) increased with structural maturity. There were significant differences in nuclear size between stages 1 and 6, as well as between stages 1 and 5, stages 2 and 6, and stages 3 and 6 (Kruskal-Wallis Dunn's multiple comparison test between animals, *P < 0.05; stage 1: n = 5 animals, stage 2: n = 7, stage 3: n = 4, stage 4: n = 6, stage 5: n = 9, stage 6: n = 11). (C) In BrdU/Prox1+ DGCs, nuclear size increased gradually with age until it reached a plateau at 35 dpi (n = 3 per group). (D, E) The majority of newborn DGCs was positioned in the SGZ and the inner half of the granule cell layer (GCL), regardless of structural stage and age. Error bars represent SEM. Scale bars in (A): 10μm. IML, inner molecular layer.

To introduce an additional indicator of cell maturation, we examined the relationship between a cell’s degree of maturation with the size of its nucleus. Since Prox1 is a specific marker for granule cell nuclei, the labeling was used to determine the nuclear sizes of DCX+ DGCs of different stages according to the DCX staging system. Fig 4A depicts 21-days-old Prox1/DCX+ cells at stage 1 (upper panel) and stage 6 (lower panel). Our results indicate that nuclear size increased with each consecutive stage (Fig 4B). DCX+ DGCs that were classified as stage 1 had on average the smallest nuclei, while stage 6 cells had the largest (stage 1: 11.79 ± 1.06 μm², n = 5; stage 2: 17.23 ± 1.05 μm², n = 7; stage 3: 21.05 ± 1.49 μm², n = 4; stage 4: 24.15 ± 1.97 μm², n = 6; stage 5: 30.56 ± 2.75 μm², n = 9; stage 6: 42.08 ± 3.48 μm², n = 11). Statistical analysis revealed significant differences in nuclear size between stages 1 and 6 (Kruskal-Wallis Dunn's multiple comparison test, P < 0.05), as well as between stages 1 and 5 (P < 0.05); stages 2 and 6 (P < 0.05); and stages 3 and 6 (P < 0.05). Subsequently, we investigated whether the nuclear size of DGCs also related to cell age. We found that nuclear size was smallest at 7 dpi (18.75 ± 0.61 μm²) and continued to increase with age until 35 dpi (47.03 ± 1.54 μm²) when it reached a plateau (Fig 4C). Consequently, our data indicate that the size of a DGC nucleus could be used as a distinct marker of cell maturation and age.

**Fig 5:**
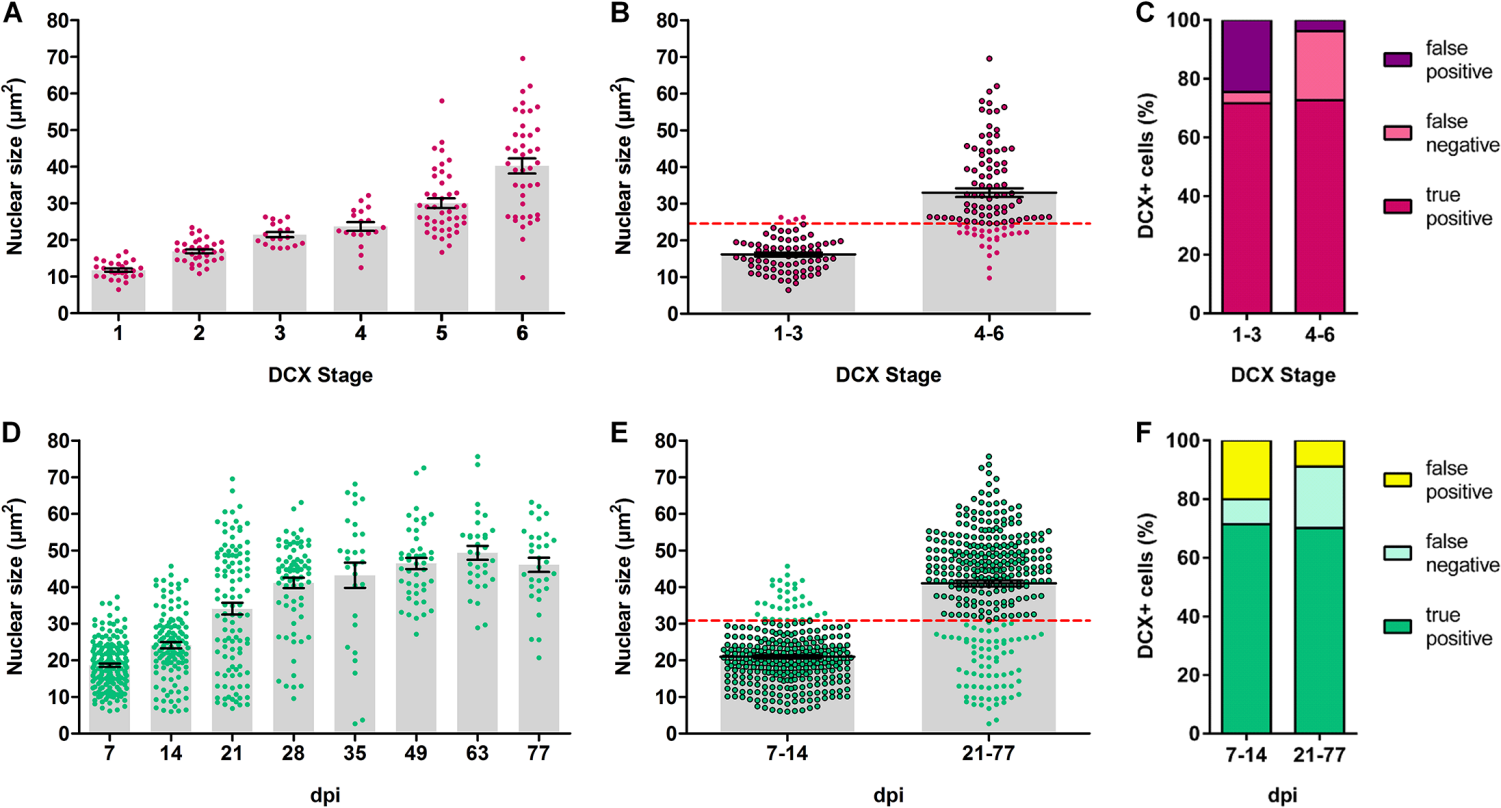
Nuclear size measurement as a valuable tool to discriminate between early and late stages in structural maturation and age of newborn DGCs. (A, D) The correlation of nuclear size with structural stage and cell age is illustrated as a dot plot (each dot represents a single cell, pooled from all animals per group) to highlight the variability. (B, E) Newborn DGCs were pooled in an early (DCX stage 1–3, cell age 7–14 dpi) and a late phase (DCX stage 4–6, cell age 21–77 dpi). The mean nuclear sizes of each group were determined and used to calculate the equidistance between early and late phases, which was then used as a threshold to discriminate and assign newborn DGCs to the early or the late phase of development (shown as red dashed line). (C, F). Based on that threshold, cells were categorized into true positive, false positive and false negative predictive values. True positive classifications were found with a reliability of about 70% across all stages. Number of animals: (A, D) DCX stage 1: n = 5, stage 2: n = 7, stage 3: n = 4, stage 4: n = 6, stage 5: n = 9, stage 6: n = 11; cell age: n = 3 per group. (B, E) DCX stage 1–3: n = 8, stage 4–6: n = 12; cell age 7–14 dpi: n = 6, age 21–77 dpi: n = 18. Error bars represent SEM.

Next, we wanted to know to what extent the nuclear size measurement of a given DGC can predict its structural (DCX) stage and cell age. Nuclear size data from all animals were pooled and are shown as a dot plot of single cells in Fig 5A and 5D to highlight the variability of the nuclear size in DCX stages and across all cell ages. The data were further pooled into an early and a late structural phase and cell age (Fig 5B and 5E), considering DCX stage 1–3 and 7–14 dpi as an early phase and DCX stage 4–6 and 21–77 dpi as a late phase. The mean nuclear sizes of each phase were determined (DCX stage early phase 16.16 ± 0.53 μm², late phase 33.00 ± 1.20 μm²; early cell age 20.95 ± 0.47 μm², late cell age 41.00 ± 0.84 μm²) and used to calculate the equidistance between early and late phases (DCX stage: 24.58 μm², cell age: 30.97 μm²), which was then used as a threshold to discriminate between early and late phases (shown as red dashed lines in Fig 5B and 5E). Based on that threshold, single cells were classified into 3 different types of predictive values: true positive, false negative and false positive (Fig 5C and 5F, see also methods for details). The amount of true positive classified cells and therefore the likelihood of a true prediction was about 70% (DCX stage 1–3: 71.57%, 4–6: 72.64%; age 7–14 dpi: 71.43%, 21–77 dpi: 70.11%).

In order to validate the prediction efficiency, an additional blinded study was performed (data not shown) in which the cell age of single cells were manually predicted from measuring the nuclear size. Samples for the early phase were selected from 7 and 14 dpi (animals = 6, cells = 109) and for the late phase from 21, 35, 63 and 77 dpi (animals = 11, cells = 74). The assignment of cells was based on the threshold (cell age: 30.97 μm²) calculated from the full dataset (Fig 5). The prediction from this blinded study showed a reliability of 72.5% for the early and 65.63% for the late cell age (true positive assignments). In summary, our calculations show that nuclear size measurement can be used as a tool to discriminate between early and late structural stages and cell ages with an accuracy of about 70%, indicating a new, valuable method to categorize developing DGCs.

### Doublecortin-expressing adult newborn dentate granule cells are located in the inner part of the granule cell layer irrespective of maturation stage and age

Previous studies have shown that young granule cells are primarily located in the SGZ and the inner part of the GCL closer to the hilus, while older DGCs are typically situated in the outer layers of the GCL, closer to the ML ([30,31]. Therefore, we subsequently investigated the positions of DCX+ somata within the GCL in relation to the cells' structural maturation and age. Triple stainings with Prox1, BrdU, and DCX enabled the analysis of cell position, stage and age of DCX+ granule cells. Cell position was defined as the shortest distance from the lower border of the Prox1+ nucleus to the GCL/SGZ border normalized to the thickness of the GCL (i.e. distance from the SGZ to the IML). Our results revealed that the majority of DCX+ newborn DGCs were located in the inner half of the GCL notwithstanding the current structural stage, although cells in stages 5 and 6 appeared to be more dispersed so that individual cells could be found in more distal parts of the GCL (Fig 4D). In addition, cell age did not appear to be correlated with the position of newborn DGCs between the ages 7–77 days. Most cells were located within the inner part of the GCL, close to the SGZ (Fig 4E). Hence, the relationship between age and cell position described previously [30,31] may only pertain to longer time courses or early (embryonic to early postnatal) developmental phases.

## Discussion

In the current study we examined the structural development of newly born dentate granule cells with a focus on a specific region of the dentate gyrus, namely the suprapyramidal blade of the septal hippocampus in Thy1-GFP mice. However, various structural and functional differences have been described throughout the septotemporal axis of the DG as well as between the supra- and infrapyramidal blades [32–35]. It has been shown that the rate of adult neurogenesis is greater in the septal DG, and the adult-born DGCs in that region display faster morphological and electrophysiological maturation compared with the temporal DG [32,34,35]. In addition, adult-born DGCs in the suprapyramidal blade exhibit a greater rate of survival compared with those located in the infrapyramidal blade [35]. The greater survival may be due to greater neuronal activity in suprapyramidal neurons [36]. Additional studies would be necessary to investigate and compare the course of structural maturation in the infrapyramidal blade and the temporal DG.

We demonstrated that Thy1-GFP does not co-localize with the immature neuronal marker DCX and hence labels only mature DGCs. This finding makes this mouse model interesting for studies concerning cell reconstruction and direct comparisons between immature and mature DGCs. Chemical markers such as BrdU, Prox1, and DCX were used to determine post-mitotic cell age, survival rates over time, and phase of neuronal development.

All animals were injected with BrdU at the age of six weeks, a time point when mice have reached fertility but may still be considered juvenile or adolescent until 2 months of age. Therefore, all analyzed cells were generated in relatively young animals. However, brain samples were harvested and examined at different time points while the animals were either adolescent (7–14 dpi) or young adult (21–77 dpi). As a result, there is a possibility that the transition between late adolescence and adulthood may have an effect on granule cell development. However, it is difficult to determine whether and how the degree of maturity of the animal past the age of six weeks might influence the course of development of individual newly born cells. By the age of six weeks, the brain development of the animals is completed and the hippocampal circuitry is established, but functional and behavioral studies showed differences in memory processing between juvenile (1–2 months) and early adult (2–6 months) mice [37].

We used DCX, which is expressed in cell somata as well as dendrites, to classify newborn DGCs into distinct structural maturation stages and thus examine relationships between structural maturation and cell age, nuclear size, and position within the GCL. While our results revealed a distinct correlation between the age and the structural development of newly born DCX+ DGCs, we found that the time in which individual neurons reach different levels of maturation varied to a large extent. In addition, nuclear size was positively correlated with both cell age and degree of structural maturation, suggesting that the size of the nucleus could be used as an additional indicator of cell development. Finally, we observed that cell position of DCX+ DGCs did not vary with cell age nor maturation stage because the majority of those cells were located within the inner layer of the GCL close to the SGZ. Our findings reveal essential structural characteristics of newborn DGCs that ought to be considered in functional studies of adult neurogenesis in mice.

### Survival of newly born dentate granule cells

To determine survival rates of newly born DGCs, we marked newborn cells by administering BrdU in a single injection of 200 mg/kg body weight. This particular administration protocol has been shown to yield maximal labeling of mitotic cells while posing a low toxicity risk in [6,38,39]. We combined BrdU-labeling with the granule cell marker Prox1 to allow for specific evaluation of DGC neurogenesis [28,29], excluding BrdU-labeling of newly generated interneurons, glial cells, or vascular tissue cells. Prox1 has been shown to be a specific marker for granule cells that is expressed throughout all developmental stages of DGCs including the phase of DCX expression. The transcription factor Prox1 plays an important role in granule cell differentiation and survival, particularly during the early stages of development [28,40,41].

Consistent with previous studies [6,42,43], we found that adult born DGC survival rate decreases steadily until 35 dpi and a constant number of surviving cells at 49, 63, and 77 dpi (Fig 2C). Our results are in line with previous findings that demonstrated a 70% decrease in BrdU+ cell number between 1 and 4 weeks following BrdU injection in mice. The number of surviving cells was not further reduced at 10 weeks following BrdU treatment [6]. Snyder et al. (2009) have established the rate of newborn cell loss over time in the mouse based on stereological analyses, a method that takes hippocampal volume changes into account, and enables generation of absolute cell numbers [6]. Nevertheless, our results based on relative cell numbers are consistent with this work. This further supports the notion that the first 5 weeks after mitosis represent a critical period of newborn DGC survival and development.

### Time course of adult-born dentate granule cell structural maturation varies between individual cells

The period of DCX expression encompasses the phases of development from neuronal progenitor to the phase when dendrites of maturing granule cells reach the OML and form functional synapses with entorhinal fibers, the main afferents to the granule cells [15,44]. DCX is expressed in the entire cell, including all neurites, and has been shown to be an accurate marker of newly born DGCs that acts as an indicator of adult neurogenesis and its modulation [45,46]. We developed a novel staging system and classified individual cells into six stages of structural maturation.

Whereas stage 1 and 2 cells represent progenitors and early immature granule cells of the SGZ [47], stage 3 and 4 cells exhibit dendrites that reach the inner and outer part of the GCL, respectively. These stages represent the phase when newborn DGCs typically start to receive synaptic input from axons of GABAergic interneurons [15]. The GABAergic input leads to depolarization in these cells and has been shown to play an essential role in the survival and further maturation of granule cells [48,49]. Stage 5 cells are characterized by dendrites that extend into the IML where they can establish synaptic contacts with glutamatergic associational/commissural fibers of mossy cells that project from ipsi- and contralateral hilar regions [15,50,51]. Most DCX+ newborn DGCs have reached stage 5 at 14–21 dpi which corresponds to the period of intense dendritic spine formation from day 16 post birth on [3,15,17,44]. However, we also still observed cells of stages 1–4 until 21 dpi, as well as cells of stage 3 at 28 dpi, which demonstrates individual differences in the time course of development between cells. This may also be a crucial period in which cell death occurs if newborn cells do not establish a sufficient amount of synaptic contacts. Finally, stage 6 DCX cells exhibit an elaborate dendritic tree that extends into the OML, where the main afferent synaptic input is received from entorhinal fibers [15,50]. Although the majority of stage 6 cells were discovered at 21–28 dpi, the first stage 6 cells could already be found at 7 dpi. This indicates that some DGCs develop very rapidly and may contact entorhinal fibers shortly after birth. Despite the high variability, some predictions can be estimated regarding the DCX cell age based on the DCX cell stage. For instance, about 70% of the early DCX cell stages 1–3 were found to belong to the group of DCX+ cells at 7 dpi, whereas about 90% of the late DCX cell stages 4–6 belonged to groups 14, 21, and 28 dpi.

Previous studies have presented temporal analyses of structural development of newborn DGCs [15,17]. In these studies, adult generated cells were labeled with a retroviral vector and examined at different time points following virus injection. The findings show a general line of increasing dendritic trees and maturation of newborn DGCs over time [15,17]. Zhao, et al. (2006) examined the morphological development of newborn neurons in postnatal and adult mouse brains. They analyzed the time course of dendritic and axonal outgrowth as well as spine formation and found that adult-generated DGCs show a slower developmental progression than cells born during an early postnatal period (P10). As a result, they defined four age-dependent stages of granule cell maturation: Stage A (0–3 dpi) during which polarization, migration and initial morphogenesis begins; Stage B (3–16 dpi) that represents major dendritic and axonal growth; Stage C (starting at 16 dpi) in which spines begin to be formed; and Stage D (starting shortly after spine formation during stage C) that is marked by synaptic modification and presence of mushroom spines [17]. In comparison, our results show that the level of structural maturation at any given time point is actually quite diverse. Consistent with our findings, Plümpe, et al. (2006) reported that structural maturation of DCX+ newborn DGCs varied between 3 days and several weeks and was not altered by regulators of cell proliferation [52]. It should be taken into account that our results are based on DCX immunoreactivity and may therefore differ from retrovirally labeled cells. However, DCX expression overlaps extensively with cytoskeletal proteins such as β III tubulin and is particularly present in distal parts of dendritic processes starting in early immature neurons and persisting throughout the time period of DCX expression [25]. Because our staging is based on how far the leading dendrite reaches into the GCL and ML, DCX labeling appears to be a practical fit for our analysis and thus any limitations regarding a nonhomogeneous distribution of the labeling in proximal dendrites would not distort our findings. Nevertheless, our results support the idea that the process of DGC maturation does not abide by a general temporal order but is influenced by more specific regulatory factors.

Our findings could have several implications in relation to the functional development of newly born DGCs, even though our DCX staging system is solely based on the structural extent of dendritic processes towards the different layers of the dentate gyrus, and does not specify functional stages. Recent studies revealed a number of differences in respect to the time period when newborn DGCs become integrated into the existing hippocampal network. While a few studies have shown that newborn DGCs received synaptic glutamatergic input at 2–3 weeks in the mouse [2,15] as well as in the rat [7,53,54], others have suggested that adult-generated DGCs display connectivity and become involved in behavior from 4–8 weeks on [55–59]. A recent study, in which the roles of both, young and old adult-born DGCs was examined, provided evidence that younger (3–4-week-old) adult-generated DGCs play an essential part in pattern separation between memories of very similar contexts and spaces, while older (>6 weeks of age) adult-born and prenatally-generated DGCs are important for rapid pattern completion, i.e. the prompt retrieval of existent memories through the use of partial cues [12]. This implies a switch of function as newborn DGCs get older. In addition, it has been shown that ablation of DCX+ neurons in adult mice leads to impairment in spatial learning acquisition but not in the recall of stored memories [60]. This further suggests that young granule cells make contributions to learning while they are still in the DCX-expression phase of development. However, since DCX+ cells exhibit variable stages of maturation, further investigations are needed to elucidate at which stage DCX+ DGCs get involved in behavior.

In a recent rat study, we examined the expression of immediate early gene (IEG) expression in newborn DGCs following long term potentiation (LTP) induction via high frequency stimulation [19]. We found that basal pCREB133 and zif268 expression is positively correlated to DCX-cell morphology. While the DCX-staging is solely based on morphology, it might indicate possible functional associations which could be examined in further studies. Furthermore, the results indicated that synaptic activation and integration of newborn DGCs took place only gradually starting at about 3 weeks of cell age. In addition, IEG-expression in newly generated cells could only be activated after the phase of DCX-expression. On the other hand, the process of functional maturation follows a gradual course that starts as early as 7 dpi [15]. The variability in the time course of functional maturation may be associated with morphological growth as indicated by DCX staging. The time frame in which the staging system can be applied appears to be a crucial phase of continuous structural growth, possibly in order to establish relevant connections to other cells, which is thus followed by stabilization of structure and functional integration.

### Granule cell nuclear size increases with each consecutive stage of structural maturation and cell age

Prox1 is a nuclear marker that is specifically expressed in granule cells [28,29]. Therefore it was used in combination with BrdU and DCX to selectively label and measure the nuclear size of newborn DGCs. We observed a direct correlation between nuclear size and structural maturation stage that revealed a steady increase of nuclear size with dendritic tree growth. Furthermore, nuclear size also increased with cell age until 35 dpi after which it remained stable (Fig 4B and 4C). Interestingly, the nuclear size of DGCs becomes constant at the same time as the number of surviving adult-born DGCs begins to stabilize (see Figs 2C and 4D). Overall, our findings support the notion that at 5 weeks of age, DGCs possess the necessary qualities that allow them to survive and become fully mature and functional.

Our finding of a concrete association of the size of the nucleus with the stage of dendritic maturation and the age of newborn DGCs can be used as a simple way to determine the maturational stage or age of a new DGC. Despite the variability of the nuclear size across the different cell ages, the steady increase within the first five weeks allows a discrimination between an early (7–14 dpi) and a late phase (21–77 dpi), which can be used to predict a cell’s age with a reliability of about 70%. Hence, the nuclear size measure shows a considerable reliability to predict the developmental stage and cell age of a newborn DGC (Fig 5) and could be used as a useful novel indicator of cell maturity.

### Adult-born granule cells remain located in the inner part of the granule cell layer

During the development of the dentate gyrus, granule cells migrate from the lateral ventricle and settle in the hippocampal area to form the GCL. The postnatally born cells are generated within the hippocampus, in what is to become the SGZ, and migrate a short distance to the inner part of the GCL, while the prenatally generated cells occupy the outer part of the GCL [61,62]. A recent study demonstrated an outside-in layering of the GCL based on the time of cell birth [31]. In addition, it has been suggested that the position of newborn cells could have important functional implications relating to the type of information these cells process (see [63] for review). Our results show that almost all of the adult-generated DGCs were located in the SGZ and inner part of the GCL. This indicates that age and degree of structural maturation did not affect migration and thus the position of the cells. Our findings support the notion that newborn DGC localization is determined very early and remains stable throughout development [43].

Our findings portray a detailed structural characterization of newborn DGCs in the adult mouse brain during the DCX-expression phase using the novel techniques DCX staging, and nuclear size measurement. This provides structural groundwork to be considered in future studies in which our tools could be combined with functional and behavioral techniques to further elucidate the process of adult neurogenesis. Detailed examinations of neural stem cell development and integration in the adult brain are needed to ultimately enable advancement in therapies for learning and memory disorders, as well as certain neurological and psychiatric diseases.

## Supporting Information

S1 Fig Minimal dataset for additional information. The supporting information file contains the detailed information for the statistical material (individual animals and numbers) presented in each figure. All of these data are illustrated in the figures.
(XLSX)
